# The complete chloroplast genome sequence of *Aglaia odorata*

**DOI:** 10.1080/23802359.2019.1704649

**Published:** 2020-01-10

**Authors:** Jinfeng Zhang, Yunqing Li, Yi Wang

**Affiliations:** Laboratory of Forest Plant Cultivation and Utilization, Yunnan Academy of Forestry, Kunming Yunnan, People’s Republic of China

**Keywords:** *Aglaia odorata*, chloroplast, Illumina sequencing, phylogenetic analysis

## Abstract

The first complete chloroplast genome (cpDNA) sequence of *Aglaia odorata* was determined from Illumina HiSeq pair-end sequencing data in this study. The cpDNA is 160,978 bp in length, contains a large single-copy region (LSC) of 88,146 bp and a small single-copy region (SSC) of 18,646 bp, which were separated by a pair of inverted repeats (IR) regions of 27,089 bp. The genome contains 129 genes, including 84 protein-coding genes, 8 ribosomal RNA genes, and 37 transfer RNA genes. The overall GC content of the whole genome is 37.5%%, and the corresponding values of the LSC, SSC, and IR regions are 35.5%, 31.8%, and 42.7%, respectively. Further phylogenomic analysis showed that *A. odorata*, *Cipadessa cinerascens* and *Aphanamixis polystachya* clustered in a clade in family Meliaceae.

*Aglaia odorata* is the species of the genus *Aglaia* within the family Meliaceae, native to Guangdong and Guangxi of China, and southeast Asian countries, often grows in sparse forests or shrubbery in low-altitude mountains. The flowers of *A. odorata* are used to perfume clothes (Janprasert et al. 1992). The extracts from *A. odorata* has antimicrobial properties (Joycharat et al. 2014). It has been used as a traditional herb to treat heart disease, bruises, traumatic injury, and pyresis (Kato-Noguchi et al. 2016). *Aglaia odorata* is also used as a potential herbicide (Laosinwattana et al. 2009) and insecticide (Nugroho et al. 1999). However, there have been no genomic studies on *A. odorata*.

Herein, we reported and characterized the complete *A. odorata* plastid genome (MN106246). One *A. odorata* individual (specimen number: 201807059) was collected from Puwen, Yunnan Province of China (22°35′34″N, 101°16′42″E). The specimen is stored at Yunnan Academy of Forestry Herbarium, Kunming, China, and the accession number is YAFH0012861. DNA was extracted from its fresh leaves using DNA Plantzol Reagent (Invitrogen, Carlsbad, CA, USA).

Paired-end reads were sequenced by using Illumina HiSeq system (Illumina, San Diego, CA). In total, about 28.9 million high-quality clean reads were generated with adaptors trimmed. Aligning, assembly, and annotation were conducted by CLC de novo assembler (CLC Bio, Aarhus, Denmark), BLAST, GeSeq (Tillich et al. 2017), and GENEIOUS v 11.0.5 (Biomatters Ltd, Auckland, New Zealand). To confirm the phylogenetic position of *A. odorata*, other 10 species of family *Meliaceae* from NCBI were aligned using MAFFT v.7 (Katoh and Standley 2013). The auto algorithm in the MAFFT alignment software was used to align the 13 complete genome sequences and the G-INS-i algorithm was used to align the partial complex sequences. The maximum-likelihood (ML) bootstrap analysis was conducted using RAxML (Stamatakis 2006); bootstrap probability values were calculated from 1000 replicates. *Citrus maxima* (KY055833) and *Glycosmis mauritiana* (KU949004) were served as the out-group.

The complete *A. odorata* plastid genome is a circular DNA molecule with the length of 160,978 bp, contains a large single-copy region (LSC) of 88,146 bp and a small single-copy region (SSC) of 18,646 bp, which were separated by a pair of inverted repeats (IR) regions of 27,089 bp. The overall GC content of the whole genome is 37.5%, and the corresponding values of the LSC, SSC, and IR regions are 35.5%, 31.8%, and 42.7%, respectively. The plastid genome contained 129 genes, including 84 protein-coding genes, 8 ribosomal RNA genes, and 37 transfer RNA genes. Phylogenetic analysis showed that *A. odorata Cipadessa cinerascens*, and *Aphanamixis polystachya* clustered in a unique clade in family *Meliaceae* (Figure 1). The determination of the complete plastid genome sequences provided new molecular data to illuminate the family *Meliaceae* evolution.

**Figure 1.**
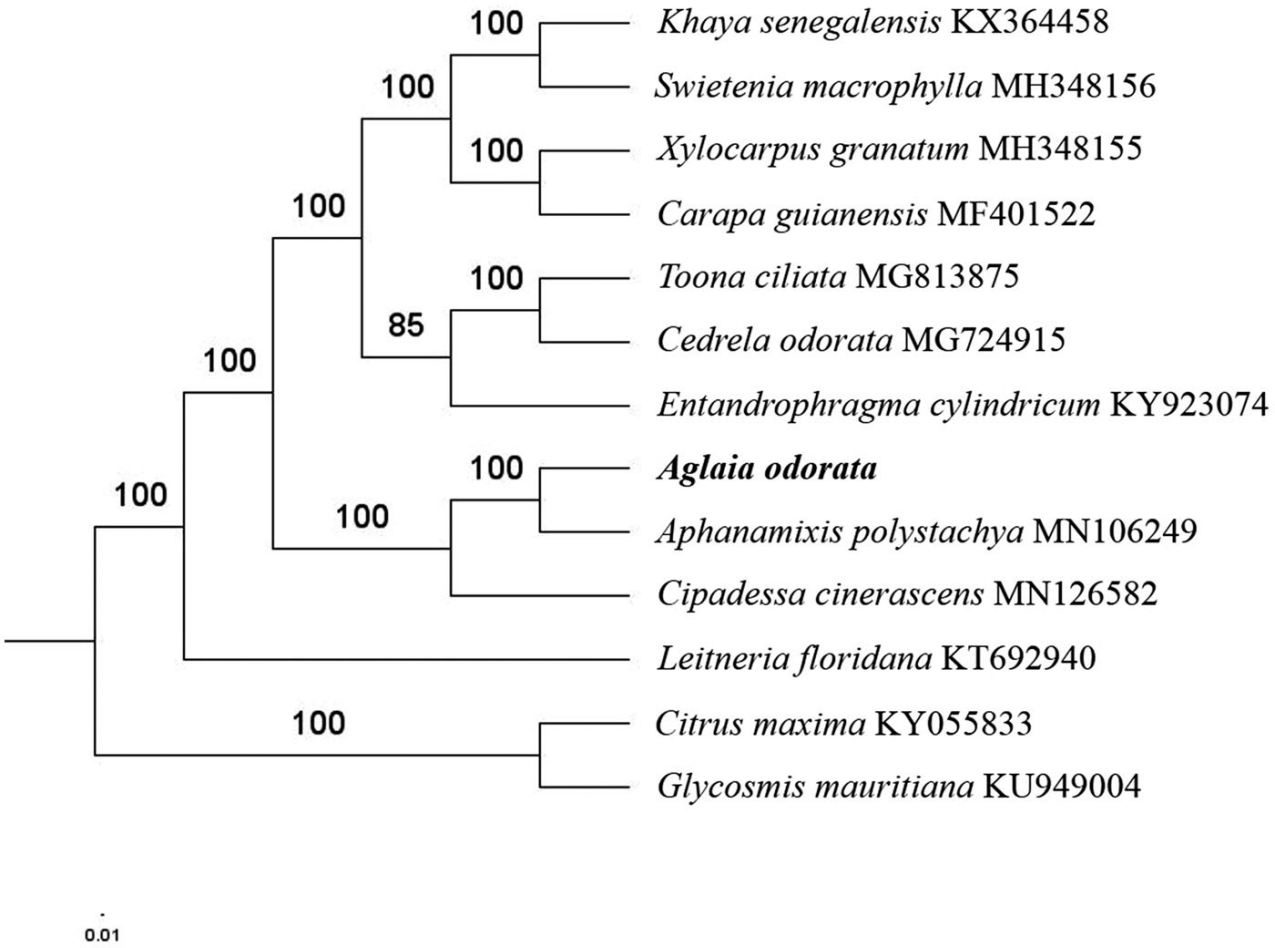
The maximum-likelihood tree based on the 11 chloroplast genomes of family *Meliaceae*. The bootstrap value based on 1000 replicates is shown on each node.
